# AMEE MedEdPublish Version 2 Launch Editorial

**DOI:** 10.15694/mep.2018.0000139.1

**Published:** 2018-07-04

**Authors:** Richard Hays

**Affiliations:** 1 University of Tasmania

**Keywords:** AMEE MedEdPublish, Opening Editorial

## Abstract

This article was migrated. The article was marked as recommended.

AMEE MedEdPublish was launched two years ago as a new outlet for scholarship in medical education ( Hays, 2016). The on-line journal format broke new ground in a context where the proportion of papers submitted to medical education journals achieving publication was falling sharply. All academic journals face challenges obtaining sufficient reviews to make sound judgements about the quality of the scholarship. Indeed, a substantial proportion of submitted papers are now rejected prior to reviews being invited. Further, while review processes are becoming increasingly open, concerns remain about the potential for various forms of bias in reviews ( Smith, 2006). Authors may be confused with editorial decisions to reject manuscripts, despite positive peer reviews. These potential helpful trails of original submission, reviews, feedback, discussion and revision are generally hidden from the readership.

MedEdPublish has taken the approach of conducting initial, ‘light touch’ reviewing by editors, rapid on-line publication and facilitating open, ‘live’ discussion between reviewers, authors and the general readership on the strengths and weaknesses of each paper. Two years after implementation, it is timely to review the initial phase, explain the revised editorial management process and provide information about the software that is being introduced with this issue.

## What have we published?

During the last two years, we have published eight themed issues at three-monthly intervals.  Themes are summarised in Table 1.  Each themed issue has had a guest Theme Editor to coordinate a series of articles related to the theme and manage other papers relevant to the theme submitted by the readership.  All themed articles are indicated by a **T** symbol in the contents pages.  Articles on topics outside the theme for the particular issue continue to be submitted, managed by the editorial team and published alongside themed papers.

A total of 593 papers have been submitted, with 512 (86.3%) accepted and 81 (13.7%) rejected.  Most decisions to reject papers were based on formatting difficulties in the management software that were able to be fixed and re-submitted.  A small number of submitted papers were judged to be not in scope or to not have appropriate ethics statements.

**Table 1.** Themes covered in the first 8 issues.

Community-based education Social and behavioural sciences in medical education Medical education in difficult circumstances Life sciences in an integrated curriculum Medical students and postgraduate trainees as educators Accessing medical education Diversity in medical education Development of health professional educators

## Reviews

Several hundred reviews were received from the panel of about 90 registered reviewers and registered readers. At least two reviews have been submitted for 76.6% of all published papers and some have received 10 or more reviews (see Table 2).  Initially, editors contributed a substantial proportion of reviews, but this is decreasing as panel and readership reviews increase in numbers. A high proportion of reviews are submitted within 72 hours of publication. The quality of reviews has varied, but most follow the guidelines for reviewers.  In general, the better papers attract higher quality reviews.  Reasons for attracting fewer reviews appear related to the format (personal opinion and editorials attract fewer responses), a narrow focus on a less popular topic, topics that may not be relevant to a larger health professions education audience, and poor readability. Another factor may be timing of publication, as papers published late in the theme cycle have high visibility for a shorter period.  Managing Editors invite further reviews where no or few reviews are submitted within four weeks of publication.

No cases of abusive reviews or responses have been reported.  While some of the discussions have been robust, the tone has remained constructive, thus we have published so far all reviews, comments and responses in the interest of openness.

There have been 20, 115, 168, 167 and 28 papers awarded, respectively, 1, 2, 3, 4 and 5 stars. The number of stars generally reflects the review activity outlined above. From a total of 512 papers submitted during the first 2 years, 193 have achieved ‘Recommended’ status. These data are summarised in Table 3.

Published articles with several reviews, responses by authors and comments from readers have provided an interesting and potentially instructive narrative. This open discussion thread should be of value in assisting educators to develop as scholars in medical education, improving their understanding of how to publish academic articles and contributing to our community of practice – scholars in health professional education.

The revised management software is being used from the current issue, commencing July 2018. While most improvements have been at the ‘back end’ to improve the editorial management process, the readership view is ‘re-freshed’, with more papers listed by recency and format. Search functions have improved. Authors will notice differences during submission. An appropriate ethics statement has become a mandatory field for papers that report research and evaluation activity, and authors are advised strongly to nominate at least two (up to four) potential reviewers, based on expertise but unrelated to the authors’ institutions and immediate professional networks.  Papers submitted without reviewer nominations will be accepted, but the review process is likely to be slower and achievement of a meaningful star rating more difficult. We are also introducing tighter timelines for themed issues and delaying publication in the final weeks of each issue, because we have noticed that papers published late in each issue are visible for shorter time and thus attract fewer reviews.

**Table 2.** No of reviews per published paper

Number of reviews received as at 31/05/2018	
0 ^*^	14
1-2	251
2-5	209
6-10	36
>10	2
Total	512

^*^ Papers just published and awaiting reviews

**Table 3.** Mean review star ratings for papers published 1/06/2016 - 31/05/2018

Mean Star rating	
0 ^*^	14
1	20
2	115
3	168
4	167
5	28
Recommended	193
Total	512

^*^ Papers just published and awaiting reviews

## Measuring the impact

Two years is not sufficient time to gain an accurate sense of the impact of the new journal on scholarship in health professions education, but there are some early signs of strong dissemination.  There have been 224,991 unique page views and 132 citations of 92 articles identified in Google Scholar.   The latter provides a strong platform on which to build citation impact once listing in indexing services is achieved.

## Where to next?

The priority for the next year is to gain recognition in indexing services, such as PubMedCentral. The initial goal of achieving listing of papers awarded ‘Recommended’ status remains. This means that we will focus on the quantity and quality of reviews as we submit our case to indexing services. Other changes to the software are being considered for the next version. Depending on how well the author nomination of reviewers’ works, this may become mandatory. Connecting themed papers across issues is a priority, as what constitutes an ‘issue’ is a rather arbitrary decision around three monthly periods, whereas many conversations should be, and are, ongoing. We are considering introducing two parallel star ratings, one each for Panel members and the general readership, to separate those reviews and potentially generate more discussion. All changes are aimed at improving the openness and quality of communication about scholarship in medical education within our community of practice and encourage ongoing scholarly dialogue.

In conclusion, after the successful, initial two years of this new AMEE journal, the future looks bright.  We invite more colleagues to participate through submitting articles, reviewing and joining in the discussion threads around each paper.

## Take Home Messages

This paper is an introduction to Version 2 of AMEE MedEdPublish Website and a review of the past two years of the publication.

## Notes On Contributors

Richard Hays is Professor of Medical Education with appointments at James Cook University and the University of Tasmania, both in Australia. He was a rural medical generalist in northern Queensland before becoming a teacher and education researcher, gaining further qualifications in educational psychology and medical education. He has had roles in the development of several medical education programmes, including new medical schools in Australia (JCU), the United Kingdom (Keele), Ireland (Limerick), Canada (Northern Ontario and Northern British Columbia) and in South-East Asia. He has also participated in many international medical education quality assurance reviews in Australia, New Zealand, the Western Pacific region, Asia, Europe and the UK. He has a strong record of gaining research grants and publishing, primarily on assessment, curriculum design and educational quality assurance (see orcid.org/0000-0002-3875-3134). He has continued part-time clinical practice throughout his career.

## Declarations

Professor Richard Hays is the Editor of the journal AMEE MedEdPublish.
