# Supplementary material for: AMEE MedEdPublish Version 2 Launch Editorial
Source: MedEdPublish (2016). 2018 Jul 4;7:139. [Version 1] doi: 10.15694/mep.2018.0000139.1 (PMC10701843; doi:10.15694/mep.2018.0000139.1)
Supplement: Peer review history

# Reviewer report: Reviewer response for version 1

**DOI:** 10.21956/mep.19606.r29125

**Published:** 2018-07-07

**Authors:** P Ravi Shankar

**Affiliations:** 1 American International Medical University

This review has been migrated. The reviewer awarded 5 stars out of 5 I read with great interest the editorial by Dr Hays about AMEE MedEdPublish. He provides a concise overview of the journal during its first two years of publishing. I am an avid reader, occasional contributor and reviewer for the journal. The journal publishes a wider range of manuscripts compared to more traditional journals. Many papers are very practical and I have learned a lot from the papers published in MedEdPublish. Articles are usually published within seven days of submission and the post publication peer review process allows for dialogue and communication between educators. These are exciting times for the journal and I wish the journal and everyone associated with it all the best and continuous growth and development. COI: I am an occasional reviewer and a member of the panel of reviewers for the journal

Reviewer Expertise:

NA

No decision status is available

# Reviewer report: Reviewer response for version 1

**DOI:** 10.21956/mep.19606.r29127

**Published:** 2018-07-06

**Authors:** Jonathan McFarland

**Affiliations:** 1 Sechenov University, Moscow

This review has been migrated. The reviewer awarded 5 stars out of 5 As the Theme Editor for the new theme on Humanities in Medical Education I read this editorial with great interest. It explained very well how far MedEdPublish has come in such a short time, giving interesting and important information on what has been published in the two years since the journal was launched. It also gives an insight into the review system, with details about the reviews per paper and the star ratings. I am very excited by this whole initiative as, for me, it is a very important step in the right direction. Richard Smith in his seminal paper, referenced in this editorial, talks about opening up the whole reviewing process, and transforming it from a black box into an open scientific discourse. I firmly believe that MedEdPublish is leading the way in this respect, and this editorial explains how this has been made to date, and what the next steps will be. I agree that the future looks bright and would suggest that this editorial is a “must read” for all those who are interested in the future of medical education. * COI - I should declare that I am Theme Editor for the theme on Humanities in Medical Education

Reviewer Expertise:

NA

No decision status is available

# Reviewer report: Reviewer response for version 1

**DOI:** 10.21956/mep.19606.r29128

**Published:** 2018-07-05

**Authors:** Sateesh Babu Arja

**Affiliations:** 1 Avalon University School of Medicine

This review has been migrated. The reviewer awarded 5 stars out of 5 This commentary is very useful and clearly explained the need for MedEdPublish. There is a strong need for a platform like AMEE MedEdPublish for the educators to disseminate their scholarly work. No only disseminating the work, there is a requirement for the platform like MedEdPublish where educators can have open access to medical education articles. This is very useful for rapid publication of articles in large number with wider access. The vision and the objectives of MedEdPublish are clearly detailed in this commentary. This is very useful for all members. All the best.

Reviewer Expertise:

NA

No decision status is available

# Reviewer report: Reviewer response for version 1

**DOI:** 10.21956/mep.19606.r29124

**Published:** 2018-07-05

**Authors:** Tripti Srivastava

**Affiliations:** 1 Datta Meghe Institute of Medical Sciences Deemed University

This review has been migrated. The reviewer awarded 5 stars out of 5 AMEE MedEdPublish has established an optimistic and welcome approach to scholarships in education which is much evident from the author’s analysis of past two years of the websites’ meaningful existence. The editorial gives a clear insight into what the team aims for MedEdPublish in near future and their strong commitment to encourage visibility of enormous work and scope in medical education worldwide. It is indeed an encouraging platform to patronize the efforts of various contributors which otherwise would have been lost in oblivion. As a review panel member, it has helped me stay abreast with the new ideas , viewpoints, trending issues and learn a bit from every manuscript. Looking forward for a rewarding experience with the new and engaging features ! All the Best for future endeavors !

Reviewer Expertise:

NA

No decision status is available

# Reviewer report: Reviewer response for version 1

**DOI:** 10.21956/mep.19606.r29126

**Published:** 2018-07-04

**Authors:** Barbara Jennings

**Affiliations:** 1 Norwich Medical School

This review has been migrated. The reviewer awarded 5 stars out of 5 In another landmark editorial for MedEdPublish, Richard Hays summarises some of this online journal's achievements and innovations. These include the handling of 593 manuscripts and 8 successful themed-editions so far; and the continuous improvement of the management software. I have just had a browse of the newly launched site and I found the layout intuitive and I love some new features; for example, I can now open a list of all the manuscripts that I have submitted reviews for. This will be a valuable curation tool for active reviewers.The ethos and practicalities of open peer-review are explored and it is encouraging to note how constructive the process has been. The impact of this journal is set to grow and grow, with PubMed indexing being the next priority for the MedEdPublish team. In the meantime we can all enjoy interesting open-access papers and more collegiate discussion about educational research.* COI: I should declare that I am a member of the journal's editorial board as well as a regular reviewer & avid reader.

Reviewer Expertise:

NA

No decision status is available
